# Intervention Development for Tailored Education for Aging and Cognitive Health (TEACH) for Dementia Prevention in Midlife Adults: Protocol for a Randomized Controlled Trial

**DOI:** 10.2196/60395

**Published:** 2024-10-16

**Authors:** Laura E Korthauer, Rochelle K Rosen, Geoffrey Tremont, Jennifer D Davis

**Affiliations:** 1 Department of Psychiatry and Human Behavior Alpert Medical School of Brown University Providence, RI United States; 2 Department of Psychiatry Rhode Island Hospital Providence, RI United States; 3 Center for Behavioral & Preventive Medicine The Miriam Hospital Providence, RI United States; 4 School of Public Health Brown University Providence, RI United States

**Keywords:** health behavior change, dementia prevention, Alzheimer disease, multidomain health intervention, intervention development, dementia

## Abstract

**Background:**

A total of 12 modifiable risk factors account for 40% of dementia cases globally, yet population adherence to health behaviors associated with these factors is low. Midlife is a critical window for dementia prevention, as brain pathology often begins to accumulate years or decades before the onset of symptoms. Although multidomain behavioral interventions have been efficacious in reducing the risk of cognitive decline, adherence is low. Intrapersonal factors, such as health beliefs, are known mediators of the relationship between knowledge and health behavior.

**Objective:**

In keeping with stage I of the National Institutes of Health (NIH) Stage Model for Behavioral Intervention Development, this study will use mixed methods to (1) develop an enhanced health education intervention, including an explanatory method for communicating information about dementia risk and personal health beliefs, and (2) conduct a pilot randomized controlled trial (n=20 per intervention arm) over 8 weeks to assess the feasibility of delivering the enhanced intervention versus basic health education alone.

**Methods:**

Phase 1 will involve focus groups and individual qualitative interviews. Focus groups will be analyzed using (1) a descriptive framework matrix analysis and (2) interpretive data review by the research team. Individual qualitative interviews will be coded using applied thematic analysis using a phenomenographic approach. Phase 2 will involve a pilot randomized controlled trial. Proximal outcomes (measured at baseline, 4 weeks, and 8 weeks) include the perceived threat of Alzheimer disease, dementia awareness, and self-efficacy.

**Results:**

This project was funded in August 2022. Data collection began in 2023 and is projected to be completed in 2025.

**Conclusions:**

Study findings will reveal the feasibility of delivering an 8-week multidomain health education intervention for primary prevention of dementia in midlife and will provide preliminary evidence of mechanisms of change.

**Trial Registration:**

ClinicalTrials.gov NCT05599425; https://clinicaltrials.gov/study/NCT05599425

**International Registered Report Identifier (IRRID):**

DERR1-10.2196/60395

## Introduction

### Background and Problem

By 2060, an estimated 13.8 million people in the United States will be living with Alzheimer disease (AD), the most common form of dementia, with health care and long-term care costs exceeding US $1 trillion annually [1]. While the US Food and Drug Administration (FDA) has approved the first disease-modifying therapies for AD, these are currently only indicated for patients in the symptomatic phases of the disease and remain costly. Primary prevention efforts are critical to achieving reductions in risk for dementia.

The 2020 report of the Lancet Commission on dementia prevention described 12 modifiable risk factors that account for 40% of dementia cases worldwide (including AD and related dementias [ADRD])—depressed mood, diabetes, early life education, excessive alcohol consumption, hearing impairment, hypertension, obesity, physical inactivity, smoking, social isolation, toxin exposure (particularly air pollution), and traumatic brain injury [2]. Many of these factors confer particular risk in midlife, when cerebrovascular changes, AD neuropathology, and related pathologies begin to accumulate in the brain [3]. Thus, targeting prevention efforts to adults in midlife or early late life is likely to confer maximal benefit [4].

Unfortunately, population adherence to health behaviors for dementia prevention is low among midlife adults. In 2014, only 28.4% of Americans aged 45 to 64 years met federal guidelines for aerobic exercise and 17.6% met guidelines for both aerobic and muscle-strengthening activity [5]. Adherence to healthy diet recommendations is also poor, with only 23.5% of Americans eating the recommended 5 servings of fruits and vegetables daily and more than 70% exceeding dietary guidelines for sodium, saturated fat, and added sugars [6]. Modification of these health behaviors through multidomain lifestyle intervention may promote positive cognitive and brain health outcomes. For example, the Finnish Geriatric Intervention Study to Prevent Cognitive Impairment and Disability (FINGER) was a multidomain invention that included physical activity, nutritional guidance, cognitive training, and management of vascular risk factors [7]. After 2 years of intervention, participants in the active treatment condition showed a 25% larger improvement in composite cognitive measures and a lower risk of cognitive decline compared to the control condition [8]. This has led to large, multisite replication studies in the United States (Alzheimer's Association US Study to Protect Brain Health Through Lifestyle Intervention to Reduce Risk [US POINTER] [9]) and worldwide, though results from these trials are not yet available. Similarly, the Systematic Multi-Domain Alzheimer Risk Reduction Trial (SMARRT) trial demonstrated that individual health coaching and nurse visits modestly improved cognition and health indices associated with dementia risk in older adults at elevated risk for dementia compared to a health education control [10].

Despite promising results of single- and multidomain intervention trials [11-13], adherence to these interventions remains problematic even with intensive individualized coaching. For example, only 19% of participants adhered to all components of the FINGER intervention (defined as attending at least 66% of sessions within each component) [14]. As expected, participants with higher adherence showed the greatest cognitive benefits of the intervention [15]. Adherence is likely to be even lower for less intensive interventions and over longer follow-up intervals. Thus, although research has identified critical components of health behavior interventions for dementia prevention, new approaches are needed to sustain health behavior change over years to decades.

### Theoretical Approach

Numerous theoretical models describe mechanisms of health behavior change. One of the oldest is the Health Belief Model (HBM) [16], which states that personal health beliefs including perceived threat of disease, perceived benefits and barriers, and self-efficacy are mediators of health behavior change. These health beliefs, as well as cues to action, motivate health behavior change. The HBM has rarely been applied to dementia directly. In 1 recent study surveying Chinese adults about knowledge of dementia prevention and current health behaviors [17], perceived benefits, cues to action, and self-efficacy played a partial mediating role between knowledge and health behavior, supporting the use of the HBM in the context of dementia prevention.

**Figure 1:**
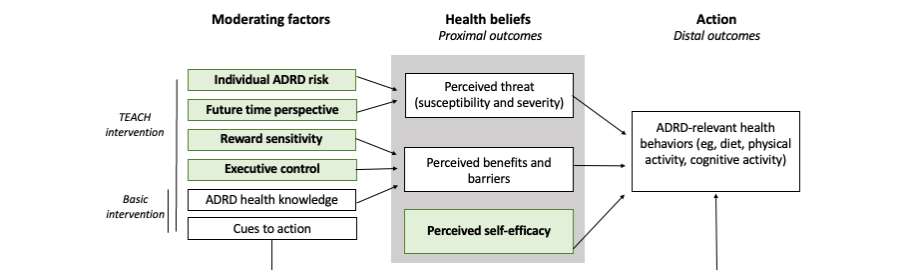
Theoretical model adapted from the Health Belief Model. Constructs shown in green reflect domains assessed via empirically validated measures from the Science of Behavior Change Research Network. ADRD: Alzheimer disease and related dementias; TEACH: Tailored Education for Aging and Cognitive Health.

We propose a working model in which individual health beliefs are moderated by constructs identified by the Science of Behavior Change Research Network [18], namely, one’s degree of dementia risk, future time perspective, reward sensitivity, and executive control moderate health beliefs, that determine the likelihood of making a health behavior change (Figure 1). By educating patients about HBM and these personal health belief factors, we hypothesize that there will be increased engagement in behaviors known to prevent dementia. The goal of this study is to use this theoretical orientation to develop a novel, personalized educational program for primary prevention of ADRD in midlife adults—Tailored Education for Aging and Cognitive Health (TEACH).

### Study Objectives and Design Overview

As a National Institutes of Health (NIH) stage I behavioral intervention development study, the primary objectives are to establish feasibility and preliminary estimates of the efficacy of the TEACH intervention. The objectives of this study are (1) to use qualitative methods to develop an enhanced health education intervention, including an explanatory method for communicating information about personal health beliefs, and (2) to conduct a pilot randomized controlled trial (RCT; n=20 per intervention arm) over 8 weeks to assess the feasibility and preliminary efficacy of the enhanced health education intervention versus basic health education alone on ADRD risk perception, self-efficacy, and knowledge about dementia risk. To establish the feasibility of measurement for future distal outcomes, we will collect pre- and posttreatment body weight, blood pressure measurement, hemoglobin A_1c_ (HbA_1c_) and lipid panel, and physical activity or sleep quality measured by a wearable activity monitor.

## Methods

### Study Setting and Recruitment

Project TEACH will take place at Rhode Island Hospital, a primary teaching hospital of the Alpert Medical School of Brown University in Providence, Rhode Island. Participants will be recruited from the Rhode Island Alzheimer’s Disease Prevention Registry and the greater Rhode Island community. All study procedures have been reviewed and approved by the institutional review board (IRB) at Lifespan, Rhode Island Hospital’s parent organization. All study-related information will be stored securely at the study site and in password-protected databases. The RCT is registered at ClinicalTrials.gov (NCT05599425). Any modifications to the protocol that could impact the conduct of the study, potential benefit to the participant, or participant safety profile will be agreed upon by all study investigators and approved by the Lifespan IRB prior to implementation. Administrative changes to the protocol, including minor corrections or clarifications, will be documented in a memorandum and in records of the protocol version.

### Ethical Considerations

The study protocol has been approved by the Lifespan IRB 3 (IRB00000482) under protocol (1895972). All participants will be screened for inclusion and exclusion criteria by phone, and eligible participants will provide written, informed consent before participating in assessment and intervention procedures.

### Participants

Inclusion criteria were adapted from the US POINTER study, a multidomain lifestyle intervention clinical trial for AD [9]. Inclusion criteria include (1) age 45-69 years, (2) normal cognition (Minnesota Cognitive Acuity scale >52 [19]), (3) proficiency in written and spoken English, and (4) at least 2 dementia risk factors that include BMI >24.9, systolic blood pressure >100 mm Hg, low-density lipoprotein (LDL) cholesterol >115 mg/dL, (4) HbA_1c_ >5.6%, (5) at least 1 apolipoprotein E ε4 allele, and first-degree relative with AD. Exclusion criteria include (1) a history of serious mental illness (ie, schizophrenia and bipolar disorder); (2) a history of major neurologic or neurodevelopmental disorder that affects cognitive performance (eg, stroke, epilepsy, and intellectual disability); (3) current alcohol or drug use disorder based on self-report; and (4) current enrollment in an AD prevention clinical trial.

### Measures

Empirically validated measures were selected from the Science of Behavior Change Research Network to assess specific health belief constructs (Table 1). These measures were administered to a group of 177 adults aged 50 years and older to establish normative data to inform interpretation within our target population [20]. Modifiable dementia risk factors will be assessed using the Australian National University Alzheimer’s Disease Risk Index (ANU-ADRI), a self-report inventory that assesses dementia risk across multiple domains and has been validated in middle-aged and older adult cohorts [21]. All measures will be administered electronically via REDCap (Research Electronic Data Capture; Vanderbilt University) survey or computerized paradigm administered via e-Prime [22]. Data will be deidentified. Data integrity will be enforced through range checks, consistency checks, and referential data rules.

**Table 1:** Health belief assessment.

Health belief domain and measure		Method		Description		Time
**Perceived susceptibility**						
	Future Time Perspective Scale [23]	10-item self-report	Perception of the future as time-limited		5 minutes	
	ANU^a^ Alzheimer's Disease Risk Index [21]	45-item self-report	AD^b^ risk assessment including demographics, medical history, physical activity, cognitive activity, social engagement, diet, and toxic exposure		20 minutes	
**Perceived benefits and barriers**						
	Monetary Choice Task [24]	27-item self-report	Reward sensitivity or delay discounting; tendency to discount future rewards (ie, preference for small rewards received sooner vs larger rewards received later)		5 minutes	
	Deferment of Gratification Scale [25]	12-item self-report	Reward sensitivity; ability to defer gratification versus pursue immediate rewards		5 minutes	
	Consideration of Future Consequences Scale [26]	12-item self-report	Reward sensitivity; tendency to guide behavior based on short versus long-term consequences		5 minutes	
	Parametric Go-No Go Task [27]	Computer	Executive control; response inhibition		7 minutes	
	Attentional Network Test [28]	Computer	Executive control; conflict monitoring		20 minutes	
**Self-efficacy**						
	Generalized Self-Efficacy Scale [29]	10-item self-report	Belief in one’s own abilities		5 minutes	

^a^ANU: Australian National University.

^b^AD: Alzheimer disease.

### Phase 1: Protocol Development

#### Objective

The first phase of the study is to develop an enhanced health education intervention using qualitative methods. The intervention adapts an existing 24-session program (2 sessions per week for 12 weeks) that was designed to educate participants about major modifiable risk factors for dementia. The basic intervention was originally designed for patients diagnosed with mild cognitive impairment or mild dementia. It is designed to increase knowledge of AD risk factors but does not include tailored content about personal health beliefs that affect health behaviors [19]. We have adapted the basic intervention for a cognitively unimpaired population in middle age or early late life by aligning the content with the modifiable dementia risk factors included in the 2020 Lancet Commission report [2] and ensuring recommendations are appropriate for the target age group rather than the older patients with mild cognitive impairment or dementia for whom the intervention was originally designed.

The TEACH intervention will include the same didactic content as the basic health education intervention. However, a major focus of the intervention will be a discussion of their health belief profiles and how their behavioral tendencies affect engagement in and maintenance of specific health behaviors. Sessions will include a discussion of perceived risk for ADRD, perceived benefits of behavior change to mitigate ADRD risk, troubleshooting barriers to action, making specific action plans, and implementing natural reward systems to bolster self-efficacy.

#### Focus Groups

##### Structure of the Focus Groups

We will first conduct focus groups (4-5 groups of 6-8 participants) to develop content, educational strategies, and delivery methods for communicating about modifiable ADRD risk factors and the HBM and personal health beliefs. During each focus group, we will present images conveying ADRD risk factors and the HBM and individual health belief factors that affect willingness to engage in behavior change. We will present a hypothetical person’s profile across the ADRD risk spectrum and health belief measures described, rather than participants’ personal health information.

We will use purposive sampling to include diverse participants based on sex, education, and race or ethnicity. Each group will take place in a private location at Rhode Island Hospital. Sessions will be digitally recorded and professionally transcribed. A research assistant will be present in the group to take notes and record nonverbal communication and participant interactions that could be missed by using the audio recording only. We plan to have 4-5 groups of 6-8 participants, but additional groups will be added as needed to reach data saturation (ie, when no new information emerges from the group discussion). The focus groups will last approximately 1 hour and will follow a discussion guide, including probes to explore and seek clarification. The groups will be attended by 2 study investigators; 1 will serve as the facilitator who will provide an overview of the group discussion and present questions and follow-up probes.

##### Qualitative Analysis

Focus groups will be analyzed using (1) a descriptive framework matrix analysis and (2) interpretive data review by the research team [30]. A framework matrix is a process for qualitative data reduction commonly used in health services research [31,32]. This approach is particularly appropriate because we require an aggregated descriptive summary of participant responses to the images, risk factors, and health beliefs reviewed in the focus groups in order to design intervention material. One rater will chart the data into a matrix by summarizing participant comments on each of the major focus group questions. A meta-summary of all individual responses will also be included in the framework matrix. All summaries will be reviewed by the research team, who will dedicate several meetings to discussing and interpreting the data. Decisions and notes from the discussion will be tracked. This 2-step process includes descriptive and interpretive transdisciplinary review by 3 clinical psychologists and a medical anthropologist. Analysis will identify the clarity of messages, alternative ways of summarizing and displaying information, and preferences for explanatory images. This will be used to refine the explanatory framework for disclosing dementia risk and personalized health belief information. Images and language used to describe the health belief constructs will be developed from the thematic analysis of the focus groups. For example, we may visually present data in images or graphs that show the relative magnitude of personal health belief traits based on individuals’ performance on the assessment measures.

#### Individual Qualitative Interviews

##### Structure of the Qualitative Interviews

We will test the explanatory framework for communicating about personal health beliefs by conducting qualitative interviews with 10-12 individuals. Participants will complete the health belief assessments. Their personalized data will be scored and presented to them in a 30-minute individual session. A trained facilitator will complete a semistructured interview with standardized questions and follow-up probes. This interview will use a phenomenographic approach, a well-accepted qualitative research method to study variations in how people learn and understand concepts in educational and health care settings [33,34]. Phenomenography examines 2 components of learning—referential and structural [35]. The referential aspect is the global meaning of the construct being conceptualized. The structural aspect is the specific combination of features (eg, images and words or phrases) being deployed.

Questions will be constructed to allow participants to reflect on their experience and will emphasize the relationship between the participant and the presented material (ie, phenomenon). The interview will include questions about acceptability (eg, “What is your reaction to your personalized health belief profile?”), appropriateness (eg, “Explain your understanding of the presented information”), and applicability (“How does this information apply to you and your health?”).

##### Qualitative Analysis

Interviews will be coded using applied thematic analysis [36]. Interview transcripts will be coded by 2 team members (a clinical psychologist and medical anthropologist) who will together discuss interview passage interpretations and apply codes attending to both the referential and structural phenomenographic meaning. Agreed-upon codes will be entered into NVivo software (version 12; Lumivero) for analysis [37]. Descriptive data summaries will be written along with interpretive qualitative memos that identify phenomenographic meaning-making by participants as they respond to intervention content and their personalized data scores [30]. Summaries and memos will be used to identify the overall preference for the presentation of data about ADRD risk. Based on these analyses, intervention content and images will be modified to ensure participant understanding, generate relevant explanations, and simplify content as needed.

##### Risks of Disclosing Personal Health Information

There is potential for disclosure of ADRD risk factors and personal health beliefs to induce distress, though risk factor disclosure has previously been demonstrated to be safe and well-tolerated by most older adults [38-40]. To mitigate risk, personalized ADRD risk and health belief information will be presented by a licensed psychologist. Immediately following the health belief disclosure, participants will complete the Perceived Stress Scale (PSS) [41] and Patient Health Questionnaire-9 item (PHQ-9) [42]. Participants will receive a follow-up phone call to readminister these measures after 2 weeks.

If any participant scores >13 on the PSS (indicating moderate distress) or >4 on the PHQ-9 (indicating possible depression), the Columbia Suicide Severity Rating Scale (C-SSRS [43]) will be administered by a trained research assistant to assess suicidal ideation and intent. Should the participant endorse active suicidal ideation with a plan or intent to act, or endorse suicidal behavior (eg, a suicide or self-harm attempt or preparatory acts), the research assistant will immediately contact 1 of the study investigators (both licensed clinical psychologists) to conduct a more thorough risk assessment and make appropriate referrals for mental health treatment.

### Phase 2: Pilot RCT

#### Objective

The second phase of the study is to conduct a pilot parallel-group, 2-arm RCT with 1:1 allocation to assess the feasibility and preliminary efficacy of the TEACH intervention compared to basic health education alone.

#### Approach

##### Procedures

A total of 40 participants will complete the baseline assessment of personal health belief factors described above (Table 1). Participants will be randomly assigned to the basic health education intervention or the TEACH intervention with a 1:1 allocation using a computer-generated randomization schedule. Due to the nature of the intervention, neither participants nor course instructors can be blinded to allocation. However, participants will be blind to study hypotheses and which intervention is considered active.

Each intervention will be conducted via a Health Insurance Portability and Accountability Act (HIPAA)-compliant videoconference platform. The intervention will be delivered twice weekly for 8 weeks (see Table 2 for class topics). The intervention will begin within 2 weeks of baseline assessments. We will recruit at least 4 participants (3 in each treatment arm) into each group, with an intended group size of 6-8. Intervention content is designed to be modular, with each session focusing on a different modifiable ADRD risk factor.

**Table 2:** Intervention class topic list.

Session	Topic
1	Physical activity (aerobic)
2	Sleep
3	Nutrition
4	Substance use (alcohol, tobacco, and cannabis)
5	Physical activity (resistance training, and mind or body practice)
6	Cognitive activity
7	Diabetes
8	Social relationships
9	Hypertension
10	Stress management and positive thinking
11	Obesity
12	Depression, anxiety, and mental health
13	Traumatic brain injury
14	Medications and supplements (including medication side effects)
15	Hearing loss
16	Air pollution and toxin exposure

Prior to beginning the intervention, participants will meet individually with the instructor for their assigned condition for a 30-minute introductory session. For participants in the basic health education treatment arm, this will include reviewing information about their personal health history and orienting them to what to expect from classes. For participants in the TEACH intervention arm, this will include education about their personal dementia risk based on their health history and responses to the ANU-ADRI, as well as their health belief factors using materials developed in phase 1.

Attendance will be taken at each session and participants will be given a schedule to track their progress and to record homework or home practice. Participants who miss a session will be contacted by email or telephone to review the missed session policy and to be encouraged to attend. Posttreatment assessments will be completed within 2 weeks of the last class and will include a measure of treatment credibility and expectancy for each arm of the trial [44].

##### Treatment Fidelity and Adherence

Participants will be blind to which treatment arm they have been assigned. All intervention classes will be video recorded. These will be reviewed by a member of the study team for treatment adherence and protocol deviations using existing monitoring protocols from our prior study of the basic health education intervention [19]. Any protocol deviations will be directly addressed and remediated.

#### Outcomes

##### Feasibility Benchmarks

A primary goal of Phase 2 is to establish the feasibility of delivering the TEACH intervention. As part of the posttreatment assessment, participants will complete 7-point Likert scales assessing the understandability, satisfaction, and perceived relevance of course material. We have established the feasibility benchmarks and they are (1) attendance—participants attend at least 75% of classes (12 of 16), (2) understandability—80% of participants agree or strongly agree that the material was understandable, (3) satisfaction—80% of participants agree or strongly agree that the program was satisfying, and (4) relevance—80% of participants agree or strongly agree that the material was relevant to their personal situation.

##### Proximal Outcome Measures

The pilot RCT is designed to estimate the preliminary efficacy of the intervention on proximal outcome measures. These will be assessed at baseline, 4 weeks, and 8 weeks (study end point). Proximal outcome measures include (1) the Perceived Threat of AD Scale [45], a 7-item Likert-type scale assessing perceived likelihood, concern, and consequences of ADRD; (2) the Dementia Awareness Questionnaire [46], a measure of knowledge of modifiable ADRD risk factors and the Generalized Self-Efficacy Questionnaire [29], which measures self-beliefs to cope with demanding situations. The 10-item PSS and PHQ-9 will also be administered at these timepoints to minimize the risk of adverse events related to personal ADRD risk and health belief information disclosure. The procedures described will be used to ensure the safety of participants who endorse clinically significant distress or depressive symptoms.

##### Distal Outcome Measures

This pilot RCT is powered to detect changes in proximal outcome measures. We will also administer some measures pre- and posttreatment to establish the feasibility of assessment and they are (1) weight measurement; (2) blood pressure measurement; (3) venipuncture for HbA_1c_ and lipid panel (total cholesterol, LDL cholesterol, high-density lipoprotein (HDL) cholesterol, and triglycerides); (4) 14-item Mediterranean Diet Assessment Tool [47]; (5) Community Healthy Activities Model Program for Seniors (CHAMPS) Activities Questionnaire for Older Adults [47]; and (6) 25-item Florida Cognitive Activities Scale [48]. Participants will also be provided a wearable activity monitor (Fitbit [Google]) to assess physical health (eg, steps per day and heart rate variability) and sleep quality. All distal outcome measures will be considered preliminary and used to inform the design of a future, fully-powered RCT.

#### Power and Data Analysis

We estimate attrition at 10% based on our previous study investigating the basic health education intervention. Power was derived based on 3 proximal outcome measures that are likely intercorrelated (assumed 0.5 correlation). Assuming 10% attrition, a sample size of 36 could detect a medium effect (*d*=0.5) with a power of 0.80 and α=.05.

All participants, regardless of adherence or attrition, will be included in the primary intention-to-treat analysis. Primary analyses will use generalized linear mixed models to compare pre- and posttreatment change in proximal outcome measures by treatment arm. We will use *t* tests to assess group differences in feasibility benchmarks (attendance, understandability, satisfaction, and relevance) by treatment arm. Secondary analyses will examine the relationship between treatment dose (defined as class attendance) and proximal outcome measures. Given that this is a stage I pilot study, all inferential statistical results will be considered preliminary and used to assess the feasibility of the TEACH intervention. Distal outcomes will not be formally analyzed but will be used to determine the feasibility of assessment for future studies.

## Results

This project was funded in August 2022. Data collection is ongoing, with 26 individuals enrolled to participate in focus groups and 11 enrolled to complete individual interviews (phase 1) by the submission of this study in May 2024. Enrollment into the RCT (phase 2) is anticipated to begin in 2024, with completion of enrollment and data analysis anticipated in 2025.

## Discussion

The goal of this study is to develop a personalized, multidomain behavioral intervention to promote long-term maintenance of health behaviors for primary prevention of ADRD in midlife and establish the feasibility of delivering this type of intervention. Despite a robust body of evidence describing modifiable health behaviors that could reduce the global dementia burden by 40% [2], multidomain behavioral interventions for the primary prevention of dementia have been hampered by poor adherence [14,15]. This study uses behavioral science to educate individuals about the intrapersonal factors that promote and maintain health behavior change (ie, understanding why we behave the way we do in addition to educating people about what behaviors are optimal). We hypothesize that this personalized approach will result in higher treatment adherence and greater efficacy of a multidomain health education intervention.

Very little research to date has used personalized intervention to promote health behavior change specifically for primary prevention of dementia. One of the challenges in dementia prevention is that individuals must maintain health-promoting behaviors like dietary changes and exercise for years to avert or delay an adverse outcome (dementia) that is perhaps decades away. Thus, effective interventions must focus on the maintenance of health behavior change over the long term. One recently completed trial, the SMARRT intervention, used motivational interviewing to assess participants’ values and motivators to reduce Alzheimer risk and help them adopt specific, achievable risk-reduction steps [10,49]. Participants in the active intervention showed greater improvements in cognition, dementia risk factors, and quality of life. However, the SMARRT trial enrolled participants aged 70 to 89 years, whereas our intervention is designed to target midlife adults aged 40-69 years. Additionally, the TEACH intervention adopts the HBM and will specifically educate people about intrapersonal processes that may promote health behavior.

This stage I intervention development project aims to use a mixed methods approach to refine a multidomain behavioral health intervention for primary prevention of dementia in midlife and early late life (age 45-69 years). The first phase of the project will use qualitative methods, including focus groups and individual interviews, to develop the personalized health education intervention (TEACH). This will include the development of an explanatory method for communicating information about personal health beliefs that is perceived to be acceptable, appropriate, and applicable to participants. The second phase of the project includes a pilot RCT to examine the feasibility and preliminary efficacy of the TEACH intervention compared to basic health education on personal dementia risk perception, dementia knowledge, and self-efficacy. If successful, this study will contribute new knowledge about personalized health education for primary prevention of dementia and a framework for educating individuals about intrapersonal processes that may be barriers or facilitators of health behavior change. Results will be used to inform intervention development and design a fully powered RCT to determine the efficacy of the TEACH intervention versus basic health education alone.

## Appendix

Multimedia Appendix 1 Peer review report from the Social Psychology, Personality and Interpersonal Processes (SPIP) Study Section - Risk, Prevention and Health Behavior Integrated Review Group - Center for Scientific Review (National Institutes of Health, USA).

## Abbreviations

AD: Alzheimer disease
ADRD: Alzheimer disease and related dementias
ANU-ADRI: Australian National University Alzheimer’s Disease Risk Index
CHAMPS: Community Healthy Activities Model Program for Seniors
C-SSRS: Columbia Suicide Severity Rating Scale
FDA: US Food and Drug Administration
FINGER: Finnish Geriatric Intervention Study to Prevent Cognitive Impairment and Disability
HbA1c: hemoglobin A1c
HBM: Health Belief Model
HDL: high-density lipoprotein
HIPAA: Health Insurance Portability and Accountability Act
IRB: institutional review board
LDL: low-density lipoprotein
NIH: National Institutes of Health
PHQ-9: Patient Health Questionnaire-9 item
PSS: Perceived Stress Scale
REDCap: Research Electronic Data Capture
SMARRT: Systematic Multi-Domain Alzheimer Risk Reduction Trial
TEACH: Tailored Education for Aging and Cognitive Health
US POINTER: Alzheimer’s Association US Study to Protect Brain Health Through Lifestyle Intervention to Reduce Risk

## Data Availability

Data sharing is not applicable to this article as no data sets were generated or analyzed during this study.
