# Supplementary material for: Intervention Development for Tailored Education for Aging and Cognitive Health (TEACH) for Dementia Prevention in Midlife Adults: Protocol for a Randomized Controlled Trial
Source: JMIR Res Protoc. 2024 Oct 16;13:e60395. doi: 10.2196/60395 (PMC11525071; doi:10.2196/60395)
Supplement: Multimedia Appendix 1 [file resprot_v13i1e60395_app1.pdf]

1R21AG075328-01A1 Korthauer, Laura

PROTECTION OF HUMAN SUBJECTS UNACCEPTABLE

RESUME AND SUMMARY OF DISCUSSION: The resubmitted application proposes developing a theory-driven, mechanism-focused personalized health beliefs intervention to lower Alzheimer's Disease (AD) risks through promoting behavior change among midlife and older adults. Reviewers agreed that the application has high significance because it could provide a personalized education model to reduce AD risks during midlife and add understanding to the role of health beliefs in preventive behavior change. The application of the Health Beliefs Model to AD risks is considered innovative. The MPI investigative team is led by clinical neuropsychologists with expertise in ADRD and consists of co-investigators experienced with qualitative research methods and intervention development and testing. The resubmission was responsive to prior reviews. Other strengths of the applications include the mixed-methods approach with an RCT pilot study design to inform preliminary efficacy, well-described recruitment strategy and approach, and the inclusion of proximal indicators of target beliefs and promising exploratory measures. Reviewers also noted some weaknesses including lacking attention to the potential negative effects from being informed of AD risks, insufficient explanation for some selected measures and risk factors (e.g., self-efficacy, AD risk index), and limited diversity in the sample to test intervention applicability to culturally diverse populations. There were some differences in opinion regarding whether these weaknesses were relatively minor and addressable. Following the discussion, most of the panel concluded that this application would have a high impact.

DESCRIPTION (provided by applicant): Modifiable risk factors such as physical activity, healthy diet, stress reduction, and cognitive stimulation are associated with lower risk of Alzheimer's disease (AD) and related dementias. Population-level engagement in positive health behaviors in midlife and early late life is low, and large, multi-domain prevention trials have reported a positive impact of lifestyle modification on risk for cognitive decline. However, adherence to trial protocols is poor despite intensive individualized coaching, raising concern about generalizability and the ability to motivate sustained behavior change. The objective of this Stage I intervention development project is to use the science of behavior change to develop a theoretically grounded, personalized health education intervention that will motivate sustained health behavior change to prevent or delay the onset of Alzheimer's disease (AD). The intervention will be grounded in the Health Belief Model, a widely-used conceptual framework that posits that health beliefs including perceived threat of disease, perceived benefits and barriers, and self-efficacy (belief in one's ability to change) are mediators of behavior change. The proposed project will enhance our existing 12-session basic healthy living education program to include evidence-based assessment of health belief factors and personalized education about the role these factors play in motivating and sustaining behavior change. We will use qualitative methods to develop an explanatory method for communicating personal health beliefs (Aim 1) and conduct a randomized controlled pilot trial to assess feasibility and the effect of the enhanced intervention versus basic health education alone on proximal outcomes including AD risk perception, self-efficacy, and AD risk knowledge (Aim 2). Results will motivate future efficacy studies investigating the impact of the enhanced healthy living education intervention on distal outcomes, including engagement in positive health behaviors, lower AD risk, and prevention of cognitive decline.

PUBLIC HEALTH RELEVANCE: Modifying health behaviors like physical activity level, diet, stress, and mental activity level can lower risk for Alzheimer's disease, but many middle-aged and older adults find it difficult to sustain health behavior changes over the long term. This project will develop a new intervention that educates people about Alzheimer's disease risk factors and helps them understand how their personal health beliefs may prevent them from making long-lasting lifestyle changes. The

goal is to help people sustain health behavior changes to prevent or delay the onset of Alzheimer's disease and related dementias.

CRITIQUE 1

Significance: 1
Investigator(s): 1
Innovation: 1
Approach: 4
Environment: 1

Overall Impact: The objective of this project is to determine whether incorporating education about personal health beliefs into a health education program is acceptable, appropriate, and applicable for healthy midlife adults who are at increased risk for AD. This project has the promise of being able to provide personalized education to middle-aged adults about their lifestyle factors and health beliefs as one way to promote AD health education during midlife. This project will involve multiple components: focus groups, qualitative interviews and RCT to test its feasibility. The project carries significance given that an increasing number of individuals will develop AD and midlife is an important stage in the lifespan to intervene in hopes of preventing or delaying the onset of AD. Innovative aspects include the mixed methods approach, focus on personalized health education, and application of the Health Belief Model to studying AD. The investigative team has the skills and expertise to carry out the goals of the project. Weaknesses of the project include lack of recruitment of a diverse sample, which will limit generalizability. The project proposed, as described in the application, promises to have a moderate to high public health impact.

1. Significance:

Strengths

- Given the aging population, there are more individuals who will eventually develop AD and personalized health education interventions can help prevent or delay AD onset, leading to a healthier overall older adult population that strains less various resources.
- Focus on midlife, given that midlife health foreshadows health in old age.
- Focus on health behaviors that can be modified or adhered to.

Weaknesses

- None noted by reviewer.

2. Investigator(s):

Strengths

- The necessary expertise is present within the team to carry out the goals of the project.

Weaknesses

- None noted by reviewer.

3. Innovation:

Strengths

- Intervention focuses on personalized education.
- Aim is to better understand why and how people engage in AD-relevant health behaviors.
- Application of Health Belief Model to AD.

Weaknesses

- None noted by reviewer.

4. Approach:

Strengths

- Mixed methods approach of including focus groups, qualitative interviews and an RCT pilot trial.
- Recruit participants from both an AD Prevention Registry and throughout the community, which promises to ensure generalizability of findings.
- The outcomes assessed are relevant and divided into proximal and distal components.

Weaknesses

- If time is primary barrier to healthy lifestyle behaviors, it is difficult to see in what ways can this personalized approach change this barrier.
- Sample to be recruited is primarily White (80 out of 90), which decreases generalizability.

5. Environment:

Strengths

- The researchers have the necessary components in place to carry out the project in a successful manner.

Weaknesses

- None noted by reviewer.

Study Timeline:

Strengths

- Appropriate for carrying out the goals of the project.

Weaknesses

- None noted by reviewer.

Protections for Human Subjects:

Acceptable Risks and/or Adequate Protections

Data and Safety Monitoring Plan (Applicable for Clinical Trials Only):

Acceptable

Inclusion Plans:

- Sex/Gender: Distribution justified scientifically

- Race/Ethnicity: Distribution not justified scientifically
- For NIH-Defined Phase III trials, Plans for valid design and analysis: Not applicable
- Inclusion/Exclusion Based on Age: Distribution justified scientifically

Vertebrate Animals:
Not Applicable (No Vertebrate Animals)

Biohazards:
Not Applicable (No Biohazards)

Resubmission:

- The team was responsive to the issues raised in the previous submission.

Resource Sharing Plans:
Acceptable

Budget and Period of Support:
Recommend as Requested

CRITIQUE 2

Significance: 2
Investigator(s): 1
Innovation: 1
Approach: 6
Environment: 1

Overall Impact: The proposed project addresses the question of whether an intervention guided by the Health Belief Model can appropriately modify health related beliefs about risk for dementia, knowledge about dementia, and self-efficacy. This is an important topic, and the use of a theoretical model to potentially customize lifestyle interventions to reduce dementia risk is important. If successful, the project could lead to a larger intervention study that could improve upon existing lifestyle interventions that often have very poor adherence and minimal success. The goal of reducing risk for Alzheimer's disease via lifestyle interventions is promising and the team is strong. However, enthusiasm is limited by a few factors, including the lack of attention to the possible negative effects of increasing participants' perceived risk for dementia, and some important limitations in the proposed measurements to develop a personalized health belief profile.

1. Significance:

Strengths

- The proposal addresses a very serious health problem, helping middle aged people reduce lifestyle related risk factors for Alzheimer's disease. If successful, this would have tremendous public health value.

- The use of the Health Belief Model to guide the research represents an important possible advance in the field.
- The project may lay the groundwork for studying very clinically relevant outcomes, such as improvement in positive health behaviors, reduced AD risk, and prevention of cognitive decline.

Weaknesses

- The proposal includes a very narrow range of proximal dependent variables, including AD risk perception, self-efficacy, and knowledge about dementia risk. There are also a number of distal outcomes that will be piloted to examine feasibility. There is controversy in the field about whether cognitive screening and informing asymptomatic persons about risk for dementia are useful or harmful and could increase psychological distress (e.g., see USPSTF Report, JAMA 2020). More discussion about the potential benefits and risks of increasing perceived risk for dementia, and inclusion of a measure of quality of life or distress would add greatly to the potential significance of the work.

2. Investigator(s):

Strengths

- The team of Investigators is well prepared to conduct most aspects of the proposed study. The team has considerable expertise in clinical neuropsychology and qualitative research methods to improve health related interventions.
- The team also has experience in conducting intervention research relevant to the proposed trial.

Weaknesses

- None noted.

3. Innovation:

Strengths

- The approach of using the Health Belief model is an important innovation in a field where many interventions do not have strong theoretical underpinnings.
- The use of qualitative information to guide the development of the intervention is an important innovation.

Weaknesses

- None noted.

4. Approach:

Strengths

- The research team has made a strong case that they can recruit the proposed sample.
- The modification of the proposal by adding recruitment outside of the registry is a significant improvement.
- The project includes proximal indicators of target beliefs and two of the three include measures that are promising.
- The project includes some exploratory measures that are gathered to document potential feasibility of a subsequent trial if results are promising.

Weaknesses

- There are elements of the intervention that are not well articulated. For example, it appears that the proposal suggests that successful intervention will increase perceived risk of dementia, as a factor that could drive motivation to change. But this may also create psychological stress as a negative factor. This issue deserves greater attention.
- The concept of self-efficacy and its relevance for the present study is not well articulated. The authors propose using a generalized self-efficacy scale. Self-efficacy theory proposes that efficacy expectations are generally situation specific, and interventions (e.g., anxiety treatments) generally target specific self-efficacy expectations. Use of a generalized indicator will likely not be sensitive to such a focused intervention.
- Similarly, the proposed health belief measures are generalized indicators, and include a measure of executive function. This does not match well with perceived benefits and barriers as described in the Health Belief Model.
- The ANU Alzheimer’s Disease Risk Index is proposed as a measure of health beliefs. However, this is actually a measure of objective risk for Alzheimer’s disease (with items such as self-reported diagnosis of diabetes and alcohol intake) and does not appear to address the perceived susceptibility as proposed in the Health Belief Model.
- Given these serious limitations in the proposed health belief assessment, it is difficult to see how the project can create a personalized health belief profile relevant to motivation to change lifestyle behaviors.
- In their discussion of Aim 2, the proposal clarifies some of these concerns about the proposed dependent variables for proximal outcomes. The Perceived Threat of AD scale and Dementia Awareness questionnaires appear appropriate and are specific to beliefs about AD. Concerns remain about the use of a generalized self-efficacy scale when more specific self-efficacy measures would likely be more appropriate and more responsive to intervention.
- The lack of attention to possible negative effects of the intervention, including possible psychological distress from interventions focused on increasing perceived dementia risk, is concerning, both from methodological and ethical perspectives.

5. Environment:

Strengths

- The environment is impressive and provides the necessary support for the proposed project.

Weaknesses

- None identified.

Study Timeline:

Strengths

- Appears appropriate.

Weaknesses

- None noted.

Protections for Human Subjects:

Acceptable Risks and/or Adequate Protections

- The project notes the possible risk of psychological risk in participants and has a clear plan to address this.

Data and Safety Monitoring Plan (Applicable for Clinical Trials Only):

Acceptable

- Appropriate.

Inclusion Plans:

- Sex/Gender: Distribution justified scientifically
- Race/Ethnicity: Distribution justified scientifically
- For NIH-Defined Phase III trials, Plans for valid design and analysis: Not applicable
- Inclusion/Exclusion Based on Age: Distribution justified scientifically
- These appear appropriate.

Vertebrate Animals:

Not Applicable (No Vertebrate Animals)

Biohazards:

Not Applicable (No Biohazards)

Resubmission:

- The proposal was appropriately responsive to the previous reviews.

Resource Sharing Plans

Not Applicable (No Relevant Resources)

Budget and Period of Support

Recommend as Requested

CRITIQUE 3

Significance: 3
Investigator(s): 2
Innovation: 3
Approach: 4
Environment: 1

Overall Impact: The proposed project will enhance an existing health education program that addresses modifiable AD risk factors, adding evidence-based assessment of health belief factors, plus personalized education about the role these factors play in motivating and sustaining behavior change. The goal is to determine whether incorporating education about personal health beliefs into a health

education program is acceptable, appropriate, and applicable for healthy midlife adults who are at increased risk for AD. Significance of this project is high, as dementia is a large public health problem and preventing it is very important. If insights can be gained regarding how to encourage individuals to do what is needed to prevent it, this will be very impactful. Also, the rationale for the project is strong, theory-driven, and based on strong empirical evidence. But it should be considered that middle aged adults who are excessively worried about dementia and perceive themselves to be at high risk for cognitive decline may be less motivated to engage in preventive activities due to “negative arousal.” Investigators: While the PIs don’t have experience leading an intervention, the close collaboration with the Co-I’s will allow them to learn “on the job.” This is a team that has worked together. The statistician added to the team needs to be named, however. Overall, the innovation is not outstanding, but the application of the HBM to middle aged adults with regard to AD is new and some of the psychological factors in focus are novel as well. The study approach is generally well-designed, but minor and moderate weaknesses with the design lower the overall impact somewhat. Cognitive screening and informing asymptomatic persons about risk for dementia could be harmful or increase psychological distress. In sum, developing an explanatory method grounded in the Health Belief Model to communicate personalized information about health beliefs that will motivate sustained health behavior change addresses a high need area and will have a profound impact, if successful. Small design tweaks could further strengthen the study. Also, lack of attention to structural inequities and disparities may make this individual-based intervention much more effective to those not affected by those inequities.

1. Significance:

Strengths

- Prevention of AD is an urgent public health issue and 12 modifiable risk factors account for 40% of dementias worldwide.
- The project has the potential to develop methods that increase adherence to positive and effective health behaviors in midlife.
- The rationale for the project is strong, theory-driven and based on strong empirical evidence.
- The intervention capitalizes on known mediators and moderators of health behavior change, such as perceived threat, perceived benefits, and self-efficacy.

Weaknesses

- Comment: While the focus on psychological and neurocognitive factors that moderate health beliefs, are more modifiable than structural/institutional factors, they are more likely to benefit those individuals who are not affected by structural and institutional racism, structural inequities in health care delivery for older adults, and other structurally based disparities. This is a limitation of individual behavior change approaches in general.
- Cognitive screening and informing asymptomatic persons about risk for dementia could be harmful or increase psychological distress. The study would be stronger if it included a measure of distress especially for individuals affected by inequities.
- Although increasing the perceived risk of dementia is seen as a motivator for preventive action, it could also lead to “negative arousal” distress related inaction and avoidance. The association among risk perceptions and health behavior may be moderated by the types and accuracy of those perceptions. For example, people’s readiness for action may be strongly influenced by whether deliberative and affective perceptions converge; middle aged adults who are excessively worried about dementia and perceive themselves to be at high risk for cognitive decline may be less motivated to engage in preventive or mitigating behaviors.

2. Investigator(s):

Strengths

- Korthauer, MPI is a clinical and research neuropsychologist, with expertise in cognitive neuroscience and neurocognitive assessment in Alzheimer’s disease will take co-leadership of this study.
- Davis, MPI is also a clinical neuropsychologist and clinical researcher with expertise in Alzheimer’s disease (AD) and related dementias, with experience conducting non-pharmacological interventions with people with MCI and family caregivers, will co-lead the study.
- Tremont, Co-I, also a clinical and research neuropsychologist, has expertise in the assessment and psychosocial intervention for patients and families with cognitive impairment and dementia. extensive intervention experience will be crucial in this project.
- Rosen, CO-I, Senior Research Scientist, medical anthropologist trained in behavioral medicine, has expertise in designing qualitative research for intervention development or adaptation; use of qualitative methods to develop, and assess the acceptability of, patient-centered interventions on mobile platforms, including user experiences with health information provided via the internet is an excellent fit for the intervention development phase.

Weaknesses

- MPIs Korthauer and Davis have no experience as PI of an intervention study.
- Whereas the introduction to the revised version mentions a quantitative statistics consultant, there is none to be found in the personnel list.

3. Innovation:

Strengths

- The intervention focuses on purported moderators of health behavior change that have been identified in early studies, but not addressed directly.
- The application of the HBM in this context and the focus on the moderators and mediators is novel.
- The development of a personalized health belief profile is innovative.
- Working with adults in middle age is innovative.

Weaknesses

- Many health behavior change interventions exist, and some theories (for example self-determination theory) may be more effective at motivating changed behavior. The use of the HBM itself is not terribly innovative.

4. Approach:

Strengths

- Study is informed by previous survey of 135 AD Prevention Registry participants on knowledge of modifiable risk factors for dementia, current engagement in healthy lifestyle behaviors, and facilitators and barriers to enact these behaviors.

- The applicants use a previously developed Healthy Lifestyle Education (HLE) program for MCI patients that was acceptable and linked to significantly greater reductions in depressive symptoms (CES-D) than a yoga intervention (but why?).
- The use of qualitative research to develop an explanatory method to communicate information about personal health belief factors for middle-aged and older adults is an effective first step to get stakeholder input.
- The plan for a randomized controlled pilot trial to assess feasibility and preliminary efficacy of the enhanced (HLE + explanatory method) vs. basic HLE intervention is clearly articulated.
- Use of a local AD Prevention Registry plus community-based recruitment ensures greater inclusivity.
- It is refreshing to see a study that includes rather than excludes people with many risk factors.
- The development of a personalized health belief profile should be quite engaging for participants.
- Aim 1a and 1b are very well explained.
- Actively engaging in community outreach targeting senior centers with higher membership of people from ethnically diverse groups in order to diversify the sample for focus groups. (However, this might not suffice.).

Weaknesses

- Minor: The placement of elements in the model (figure 1) suggests that perceived threat, perceived benefits, and self-efficacy are mediators of the purported moderating factors of the enhanced intervention. This is confusing.
- Minor: It would seem that a generalized self-efficacy scale would not be as relevant as a specific behavior-change self-efficacy scale.
- Minor: Is two sessions/week for 12 weeks for a HLE realistic for middle-aged (working) adults? I certainly couldn’t squeeze that in.
- Question: asking about preferred delivery method in focus group -- Can the participants try out these different methods, or are they expected to evaluate them without really experiencing them?
- Minor: “Once sessions commence, participants will be added to the ongoing basic or enhanced HLE classes as they are recruited.” Does this mean rolling enrollment? How do you ensure that participants are exposed to all intervention material, or do they just continue for 12 weeks regardless of when they started? This approach may have a negative effect on group cohesion.
- Moderate: Location of study activities. Previously it was mentioned that some individuals dropped out of the program because of transportation difficulties. In the recruitment and retention section, the distribution of detailed maps, directions, and parking vouchers will be provided (which is great). However, there is no mention of transportation assistance (for individuals with low income). Community locations are often more attractive and more accessible for people with transportation and/or mobility issues.
- Minor: Moving to zoom – when smartphones are used, there is often poor visual quality – lending out tablets may work better.
- Moderate: The focus on English speakers will exclude older Latinx groups who are not bilingual.

- It may not be helpful to include more than one non-white ethnic group, since small subgroups cannot be effectively compared, and variability introduced by small subgroups is often taken back out of the model through covarying ethnicity.
- The personalized health belief profile contains some variables that don’t really define beliefs.

5. Environment:

Strengths

- Brown University, Rhode Island Hospital, the Neuropsychology Program at Rhode Island Hospital, and The Miriam Hospital are well-resourced research environments that should easily facilitate carrying out this project

Weaknesses

- None noted by reviewer.

Study Timeline:

Strengths

- Overall appropriate but may be tight.

Weaknesses

- It is unclear whether the pilot RCT can be carried out in 9 months.

Protections for Human Subjects:

Unacceptable Risks and/or Inadequate Protections

- The focus groups may pose a risk for lack of privacy and lack of confidentiality. This needs to be addressed in the Human Subjects section.

Data and Safety Monitoring Plan (Applicable for Clinical Trials Only):

Acceptable

Inclusion Plans:

- Sex/Gender: Distribution justified scientifically
- Race/Ethnicity: Distribution not justified scientifically
- For NIH-Defined Phase III trials, Plans for valid design and analysis: Not applicable
- Inclusion/Exclusion Based on Age: Distribution justified scientifically
- It is not clear whether mere "representation" of several ethnic groups in small numbers makes sense in the small pilot clinical trial. Since with very small numbers, no meaningful cultural comparisons can be made, and thus, variation will likely be excluded by using ethnicity/race as a covariate, a more homogeneous sample or limiting oneself to only 2 ethnic groups who can be compared is the more informative approach.

Vertebrate Animals:

Not Applicable (No Vertebrate Animals)

Biohazards:

Not Applicable (No Biohazards)

Resubmission:

- The resubmission addressed most critiques. Although the addition of a statistician is mentioned, this person doesn't show up in the personnel list.

Resource Sharing Plans:

Acceptable

Budget and Period of Support:

Recommend as Requested

THE FOLLOWING SECTIONS WERE PREPARED BY THE SCIENTIFIC REVIEW OFFICER TO SUMMARIZE THE OUTCOME OF DISCUSSIONS OF THE REVIEW COMMITTEE, OR REVIEWERS' WRITTEN CRITIQUES, ON THE FOLLOWING ISSUES:

PROTECTION OF HUMAN SUBJECTS: UNACCEPTABLE
The committee noted that privacy protection and confidentiality procedure for the focus groups was inadequately described.

INCLUSION OF WOMEN PLAN: ACCEPTABLE

INCLUSION OF MINORITIES PLAN: ACCEPTABLE

INCLUSION ACROSS THE LIFESPAN: ACCEPTABLE

COMMITTEE BUDGET RECOMMENDATIONS: The budget was recommended as requested.

Footnotes for 1 R21 AG075328-01A1; PI Name: Korthauer, Laura

+ Derived from the range of percentile values calculated for the study section that reviewed this application.

NIH has modified its policy regarding the receipt of resubmissions (amended applications).See Guide Notice NOT-OD-18-197 at https://grants.nih.gov/grants/guide/notice-files/NOT-OD-18-197.html. The impact/priority score is calculated after discussion of an application by averaging the overall scores (1-9) given by all voting reviewers on the committee and multiplying by 10. The criterion scores are submitted prior to the meeting by the individual reviewers assigned to an application, and are not discussed specifically at the review meeting or calculated into the overall impact score. Some applications also receive a percentile ranking. For details on the review process, see http://grants.nih.gov/grants/peer_review_process.htm#scoring.
